# Chronic restraint stress induces excessive activation of primordial follicles in mice ovaries

**DOI:** 10.1371/journal.pone.0194894

**Published:** 2018-03-30

**Authors:** Minhua Xu, Junyan Sun, Qian Wang, Qiuwan Zhang, Chunsheng Wei, Dongmei Lai

**Affiliations:** 1 The International Peace Maternity and Child Health Hospital, School of medicine, Shanghai Jiaotong University, Shanghai, China; 2 Eye and ENT Hospital, Fudan University, Shanghai, China; China Agricultural University, CHINA

## Abstract

Chronic stress is an important factor influencing people’s health. It usually causes endocrinal disorders and a decline in reproduction in females. Although studies of both human and animals suggest a detrimental effect of stress on reproduction, the influence of chronic stress on the ovarian reservation and follicular development is still not clear. In this study, a chronic restraint stress (CRS) mouse model was used to investigate the effect of stress on ovarian reservation and follicular development and explore the underlying mechanism. In this study, after 8 weeks of CRS, primordial follicles were excessively activated in the ovaries of the CRS group compared with the control group. Further results showed that the activation of primordial follicles induced by CRS was involved in the increasing expression level of Kit ligand and its receptor Kit and the activation of phosphatidylinositol 3-kinase (PI3K)/phosphatase and tensin homolog deleted on chromosome 10 (PTEN)/protein kinase B (Akt) pathway. The corticotropin-releasing hormone (CRH) is a neuropeptide released due to stress, which plays an important role in regulating follicle development. A high level of serum CRH was detected in the CRS mouse model, and the real-time polymerase chain reaction assay showed that the mRNA level of its main receptor CRHR1increased in the ovaries of the CRS mouse group. Moreover, 100nM CRH significantly improved the activation of primordial follicles in newborn mouse ovaries *in vitro*. These results demonstrated that CRS could induce immoderate activation of primordial follicles accompanied by the activation of Kit-PI3K signaling, in which CRH might be an important endocrine factor.

## Introduction

Chronic physical and psychological stress has become a major health issue across the world. Both clinical and animal studies have confirmed that stress is an important risk or aggravating factor for several diseases, including hypertension, cardiovascular disease, bone loss, neurodegenerative diseases, and cancer [1–5]. In fact, females are easier targets of stress compared with males [6, 7]. Studies have shown that different kinds of stress altered ovarian function and follicle recruitment in animal models. For example, chronic unpredictable mild stress inhibited ovarian steroidogenesis and follicular development, causing follicular atresia in mice ovaries [8]. Chronic intermittent cold stress changed the follicular development associated with a neurotrophin-dependent sympathetic nerve activation in rats [9]. Another study demonstrated that heat stress not only had an immediate influence on the follicular dynamics of cows but also had a delayed effect on the follicular growth [10].

In mammalian ovaries, a primordial follicle pool is developed in fetal life (in human) or developed post natally (in mice or rats) (11). The dormant primordial follicle pool is a relatively fixed and limited source of developing follicles. The regulation and control of activation of primordial follicles are essential to the maintenance of normal reproductive span. If the normal balance is disrupted, those primordial follicles can be exhausted faster under the situation of abnormal and excessive activation. Multiple studies have shown that the activation of primordial follicles is regulated by many different signaling pathways [11–13], among which the Kit ligand (Kitl) is a key growth factor in the ovary. Kitl, which is synthesized and secreted by granulosa cells (GCs), directly stimulates the activation and growth of oocytes by binding to its receptor Kit. Available evidences shows that Kitl is an important positive regulator of activation of primordial follicles through the phosphatidylinositol 3-kinase (PI3K)/phosphatase and tensin homolog deleted on chromosome 10 (PTEN)/protein kinase B (Akt) pathway [14–19].

The hypothalamic–pituitary–adrenal (HPA) axis is activated under stressful situations. The corticotrophin-releasing hormone (CRH) is a neuropeptide produced by the hypothalamus during stress. CRH plays a crucial role in regulating ovarian functions, since its receptors have already been identified in female reproductive organs, including ovary, uterus, and placenta [20, 21].

A chronic restraint stress (CRS) mouse model is a commonly used model in studies related to physical and psychological stress [2, 22–24]. A CRS female mice model was established in this study to imitate the stress in modern women. The study aimed to explore the direct effects of CRS on ovarian reservation and the underlying mechanism.

## Materials and methods

### Establishment of the CRS mouse model

Female BALB/c mice aged 6 weeks were purchased from Shanghai Laboratory Animal Co., Ltd and housed in the Department of Animal Experiments, Medical School of Shanghai Jiao Tong University (Shanghai, China). The mice were randomly separated into control and CRS groups. Centrifuge tubes (50 mL) were used to generate stress, which were multi-punctured to maintain enough ventilation. The CRS group mice were placed in these tubes from 9:00 a.m. to 3:00 p.m. Both the CRS group and the control group mice were deprived of water and food during this time to diminish the confounding factor. This procedure was carried out for 6 days in a week randomly for continuous 3 weeks (the CRS 3w group) or 8 weeks (the CRS 8w group). The body weights of mice were recorded every other week. In the seventh to eighth week of CRS, vaginal smears of the control and CRS 8w groups were performed every morning to record the estrous cycles of the mice. The smears were stained with Wright’s dye and observed under a microscope. When the CRS protocol ended, the mice of different groups were euthanized after anesthesia by inhaling isoflurane (RWD Life Science Co., Ltd, R510-22). Serum and bilateral ovaries were collected for future analysis.

All procedures for animals were approved by the Institutional Animal Care and Use Committee of Shanghai (Protocol number: B-2015-016) and performed in accordance with the National Research Council Guide for Care and Use of Laboratory Animals. Efforts were made to minimize animal suffering and limit the number of animals used in the study.

### Histological evaluation

The ovaries were collected from the control and CRS groups after the CRS procedure was done. The ovaries were fixed in Bouin’s solution (containing 5% acetic acid, 9% formaldehyde, and 0.9% picric acid), paraffin embedded, and serially sectioned at a thickness of 5 μm. Hematoxylin–eosin (H&E) staining was used to evaluate the morphological structure of the ovaries, which was evaluated using a microscope. The follicles were categorized and counted in every fifth section of the ovary, using a method described previously[25, 26]. Briefly, a primordial follicle was defined as a single layer of fusiform GCs surrounding an oocyte. A primary follicle was surrounded by at least three GCs resulting in a cubic shape, and a secondary follicle appeared surrounded by at least two layers of GCs with no follicular cavity. Corpus luteum was identified with active luteal cells, which had basophilic cytoplasm with large nuclei and prominent nuclei[27].

### Hormone assays

The blood samples of the control and CRS groups were collected and centrifuged (3000 rpm, 15 min) to separate serum after standing for 30 min at room temperature (RT). The serum collected was stored at −80°C until further use.

Serum concentrations of cortico sterone [limit of detection (LOD), 0.103–2.5 ng/mL; coefficient of variation (CV), <10%; R&D, Minneapolis, USA)],CRH(LOD, 0.03–4 ng/mL; CV,<10%; Shanghai Westang Biological Technology, China), estradiol(LOD, 0.25–2 pg/mL; CV,<15%; Shanghai Yuanye Biological Technology,China),testosterone(LOD, 0.1–20 ng/mL; CV,<15%; Shanghai Yuanye Biological Technology),and AMH(LOD, 0.23–15 ng/mL; CV,<15%; Ansh Lab, texas, USA)were assayed using the enzyme-linked immuno sorbent assay (ELISA) kit and procedures provided by the respective manufacturer.

### Immunohistochemistry

Tissue sections made as described earlier were deparaffinized and dehydrated. Then, the slides were incubated in a 3% H_2_O_2_ solution to quench the endogenous peroxidase, followed by rinsing in phosphate-buffered saline (PBS). The sections were then treated with heated antigen retrieval solution containing citrate antigen retrieval solution (Beyotime, China) to retrieve the antigenicity. After being incubated with 10% goat serum/0.3% Triton X-100 in PBS for 30 min to block the nonspecific antibody-binding sites, the samples were incubated with the following primary antibodies: rabbit anti-Akt monoclonal antibody (Ser473, 1:50; CST, Massachusetts, USA) and rabbit anti-foxo3a monoclonal antibody(1:1600; CST) at 4°C overnight. Then, the sections were washed and probed with horseradish peroxidase–labeled anti-mouse/rabbit second antibodies. A peroxidase substrate was developed using a diaminobenzidine substrate kit (Wuhan Goodbio Technology, China). The slides were counterstained with hematoxylin, dehydrated through a graded ethanol series, and mounted using the PermountMounting Medium(Specimens of Harbin green technology development co., China).

### RNA extraction, reverse transcription of mRNA and real-time polymerase chain reaction analysis

The total RNA was isolated from mouse ovaries, cultured human GCs, and cultured ovaries using a TRIzol reagent (Invitrogen, CA, USA). cDNA was synthesized using the PrimeScript RT Reagent Kit (Applied Biosystems, Takara, Japan) according to the manufacturer’s instruction, using 500 ng of total RNA per each sample. Real-time quantitative polymerase chain reactions (PCRs) were performed in triplicate using the SYBR Green Real-time PCR Master Mix (Applied Biosystems, Takara, Japan) with the Mastercycler ep realplex real-time PCR system (Eppendorf, Hamburg, Germany). The PCR primers were designed according to cDNA sequences in the National Center for Biotechnology Information database. Primer sequences are listed in supporting file (S1 Table). Cycling conditions for the PCR system were as follows: 95°C for 5 min, and 60°C for 34 s for 40 cycles. The gene expression levels were evaluated using the ΔΔCT method, standardized to levels of β-actin amplification.

### Western blot analysis

Protein lysate from fresh ovarian tissue was prepared, separated on 10% sodium dodecyl sulfate–polyacrylamide gel, and transferred to a polyvinylidene difluoride membrane (Millipore, California, USA). The membranes were blocked with 5% nonfat milk in Tris–HCl (10mM, pH 7.4) containing 150mMNaCl and 1% Nonidet P-40 and separately incubated with the following specific antibodies at 4°C overnight: rabbit anti-Kitl polyclonal antibody (1:1000, ab64677; Abcam, Massachusetts, USA), rabbit anti-PTEN monoclonal antibody (1:1000, 9559; CST), rabbit anti-Aktmonoclonal antibody (1:1000, 4691; CST),rabbit anti-pAkt monoclonal antibody (Ser473, 1:1000, 4691; CST), rabbit anti-foxo3a monoclonal antibody(1:1000, 12829; CST), and rabbit anti-pfoxo3a polyclonal antibody(Ser253, 1:1000, 9466; CST). The relative intensity of protein bands was quantified by digital densitometry (ImageJ software, National Institutes of Health, Maryland, USA). The β-actin levels were used as internal standards.

### Primary granulosa cell collection, culture and cell proliferation assay

Primary granulosa cells were collected based on previously described procedures with modification[28]. Briefly, 21-day-old female mice (Balb/c) were intraperitoneally injected with 5 IU pregnant mare serum gonadotropin (Ningbosansheng pharmaceutical Co. Ltd. CA). At 46 h after injection, the follicles were punctured with a 25-gauge needle to release both oocyte-cumulus cell complexes and clumps of mural granulosa cells. The granulosa cells were collected in basic culture medium consisting of a phenol red-free Dulbecco’s modified Eagle’s medium/F-12 medium(DMEM/F12;Gino biomedical technology co., LTD, CA) supplemented with 10% fetal bovine serum(FBS; Life Technologies,waltham, MA, USA),1xITS (5 lg/ml insulin, 5 lg/ml transferrin, and 5 lg/ml selenium) (Sigma-Aldrich, St. Louis, MO, USA), 50ug/ml sodium pyruvate (Gibco, waltham, MA, USA), 100 U/mL streptomycin and 100 U/mL penicillin (Gibco, waltham, MA, USA). Cells were dispersed by gentle drawing in and out of a pipette and then centrifuged for 3 min at 250xg. The granulosa cells were resuspended in basic culture medium and seeded in 6-well plates at a density of 5x10^5^ cells/well and cultured for 24 h in a humidified atmosphere containing 5% CO2 at 37°C. Then, the culture medium was changed with basic culture medium or the conditional medium supplemented CRH (Sigma-Aldrich, St. Louis, MO, USA)1nM,10nM,100nM. After 48h culture, the cells were harvested for PCR assay.

The cells were plated at a density of 2×10^4^ cells/well on 96-well plates in 100ulbasicculture medium. The cell number was determined by the WST-8 [2-(2-methoxy-4-nitrophenyl)–3–(4-nitrophenyl)–5—(2,4-disulfophenyl)–2H–tetrazolium monosodium salt] assay using a Cell Counting Kit 8 (CCK-8) [Yeasen Biotechnical Company (40203ES76)] according to manufacturer’s instructions.

### In vitro culture of mouse ovaries

Ovaries of 3-day-old female BALB/c mice were isolated and cultured (37°C, 5% CO_2_) on Transwell Permeable Supports (Corning, NY, USA) in the DMEM/F12 medium (Invitrogen, CA, USA) supplemented with 10% FBS (Life Technologies, waltham, MA, USA) 0.23mM pyruvic acid, 3 mg/mL BSA (AMRESCO LLC, Texas, USA), 1×Insulin, Transferring, Selenium Solution(ITS) supplement (Invitrogen, CA, USA), and 1× Antibiotic-Antimycotic (Invitrogen, CA, USA). Moreover, CRH (TocrisBristol, UK) was added into the medium of CRH group ovaries, making a final concentration of 100nM. The medium was changed every other day. After 7 days of culture, ovaries of the control or CRH 100nM group were collected for RNA extraction or morphological analysis.

The developing follicles were defined as follicles with a diameter of oocytes greater than 20μm, which were selected into a growing follicular pool relative to the quiescent primordial follicles, containing small oocytes (>20 μm) surrounded by flat GCs[15].

### Statistical analysis

All data were expressed as mean ± standard error of mean. Statistical significance was determined by the GraphPad Prism software (GraphPad Software Inc., California, USA). The differences in the body weight and the GCs viability in different CRH concentrations in vitro varied with time were analyzed by Repeated Measures(RM). And, the difference in serum hormone level, gene expression, and protein profile between the control and CRS 8w groups were evaluated using the unpaired two-tailed Student *t* test. Besides, data of follicular number, ratio of primary follicles/primordial follicles among control, and CRS 3w and CRS 8w groups were obtained using the one-way analysis of variance(ANOVA) following the Tukey post-hoc test. In the *in vitro* experiment, the optical density value and the expression of kitl were assessed using the one-way ANOVA following the Tukey post-hoc test. The *P* values <0.05 were considered statistically significant. All experiments were repeated independently at least three times.

## Results

### CRS induced body weight loss and caused deregulation of estrous cycle in mice

**Fig 1:**
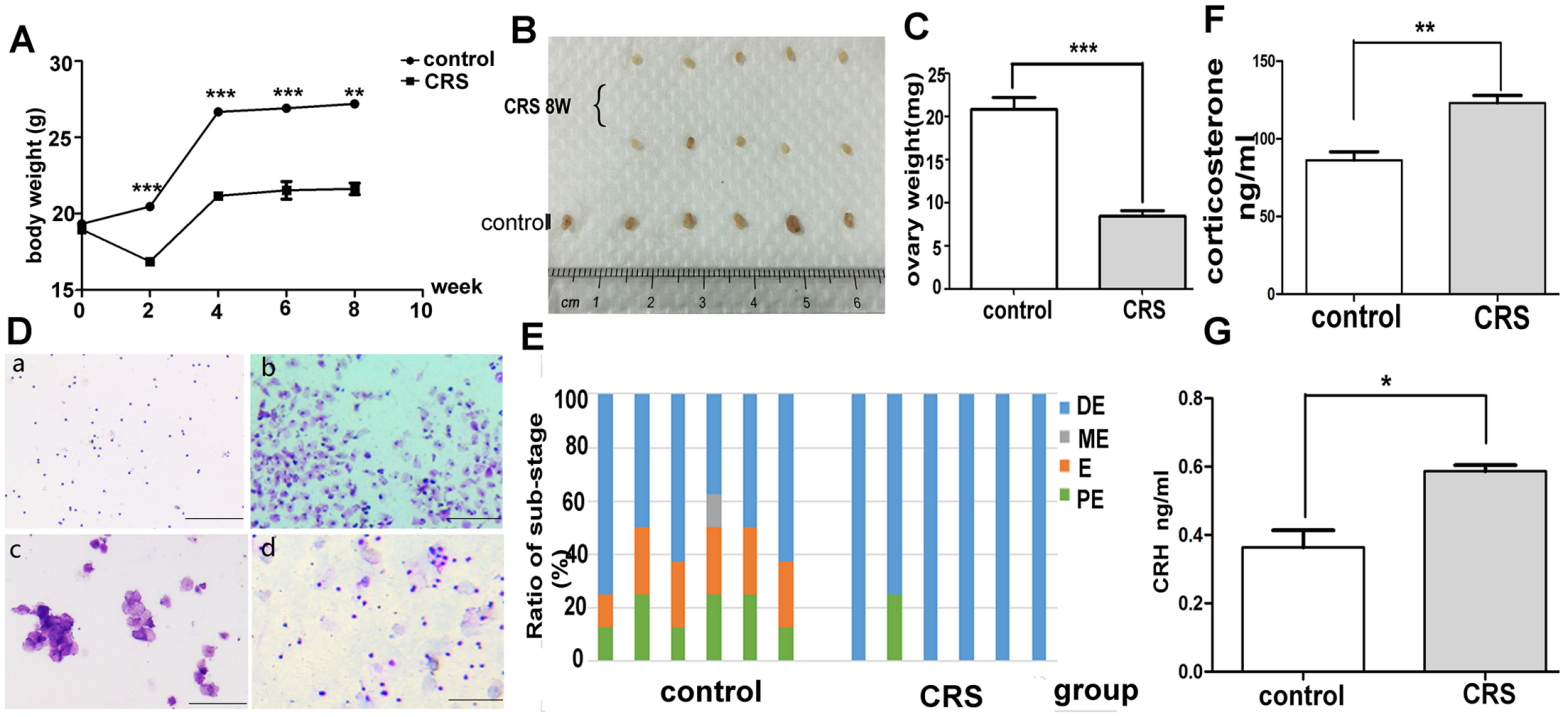
CRS reduced the body weight of mice and caused deregulation of the estrous cycle. (A) CRS reduced the body weight of mice (*n* = 10, *P*< 0.05). (B) The ovarian gross morphology of the control and CRS 8w groups. The size of the control group was almost two times larger than that of the CRS group. (C) The ovarian weight was significantly decreased in the CRS 8w group compared with the control group (*n* = 6, *P*< 0.001). (D) The morphological characterization of the smears in every substage of the mice estrum cycle. Briefly, three types of cells were present in the smear, including small leukocytes, larger and round epithelial cells, and large, flat, and nonnuclear cornified cells. The relative numbers of leukocytes (L) and epithelial (E) and cornified (C) cells in the vaginal smear indicated the stage of the estrous cycle. (a) Contained L and was classified as diestrum (DE). (b) Contained E and was classified as proestrum(PE). (c) Contained C and was classified as estrum (E). (d) Contained three types of cells and was classified as metaestrum (ME). (Bar = 100μm) (E) CRS caused deregulation of the estrous cycles of mice, and the CRS 8w group remained in the diestrum stage almost throughout the cycle (*n* = 6; PE, proestrum; E, estrum; ME, metaestrum; DE, diestrum). (F) Serum corticosterone level of mice in both control and CRS 8w groups (*n* = 10, *P*< 0.001). (G) Serum concentration of CRH in mice of both control and CRS 8w groups (*n* = 10, *P*< 0.05). CRS, Chronic restraint stress.

The CRS mice model was established in the present study to exert stress on mice. We observed that the mice in the CRS group had a significantly lower body weight each week compared with those in the control group (Fig 1A). The body weight of the CRS group decreased in the first 2 weeks and then increased slowly. The food and water intake of the mice was monitored to verify whether the CRS protocol affected the basic daily intake. The amount of intake was not significantly different between the control and CRS groups (S1A Fig), demonstrating that the loss in body weight of the CRS group was not due to lower intake. Furthermore, the ovarian gross morphology in the control and CRS 8w groups was evaluated. The size of the control group was almost two times larger than that of the CRS 8w group (Fig 1B and 1C, *P*<0.001). Also, vaginal smears were performed and morphological characterization of the smears in every substage was shown (Fig 1D). The results showed that all mice in the CRS 8w group lost the regular pattern of estrous cycle. The CRS 8w group stayed in the diestrus stage and lost estrum most of the time (Fig 1E). Furthermore, the concentrations of serum corticosterone and CRH were significantly higher in the CRS 8w group than in the control group after 8 weeks of CRS (Fig 1F and 1G). The levels of serum estradiol and testosterone were also tested. The results showed a slight reduction in both hormones in the CRS 8w group, but no significant difference was found compared with the control group (S1B and S1C Fig).

### CRS treatment induced abnormal activation of primordial follicles and upregulation of AMH in mice

**Fig 2:**
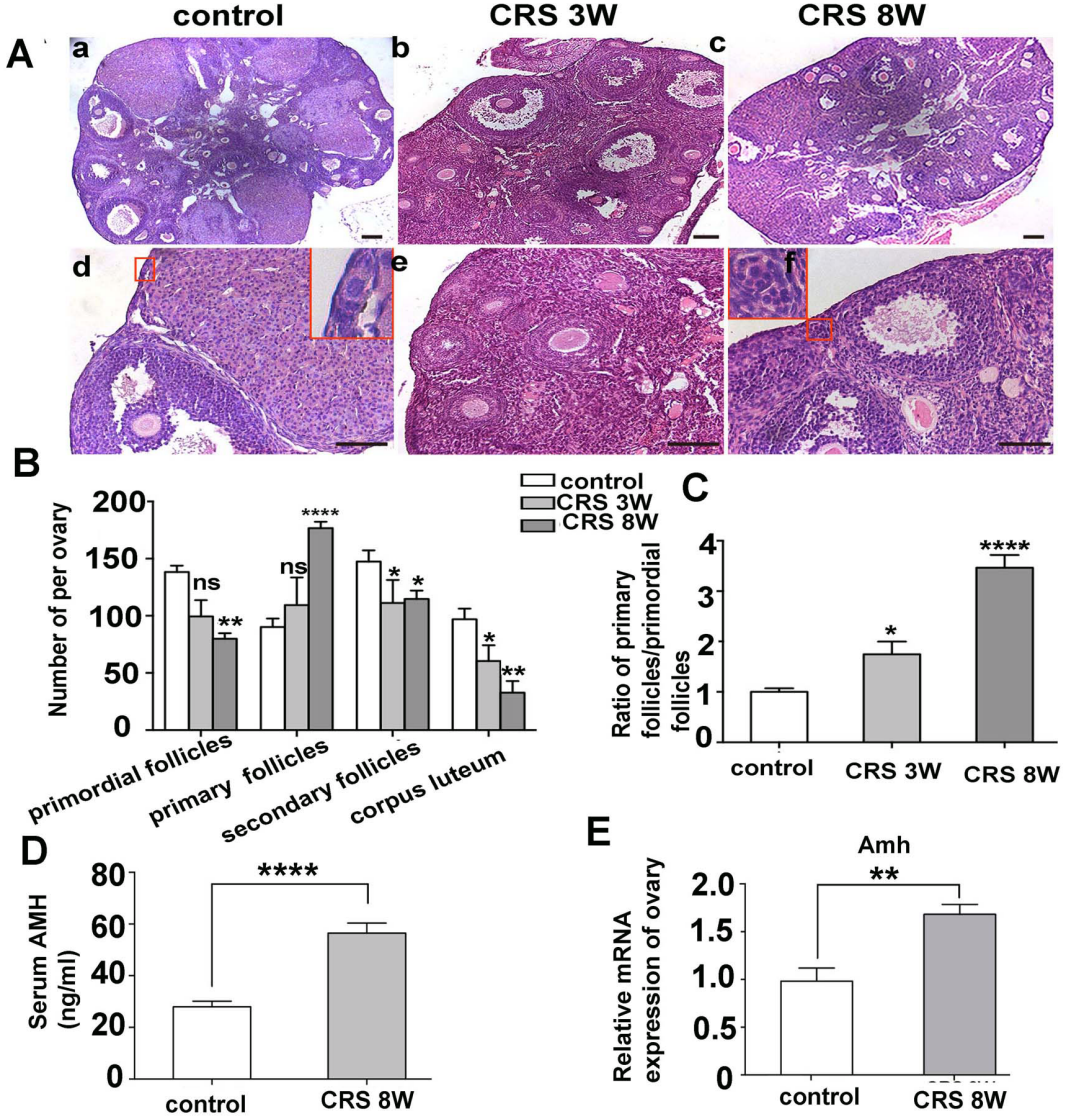
CRS induced activation of primordial follicles and up-regulated the level of AMH. (A)H&E staining morphological features of ovaries derived from the control and CRS groups (scale bar, 100 μm). The red outline showed the enlargements of primordial follicles (d), primary follicles (f) and secondary follicles(b). (B)Effects of 3 or 8 weeks of CRS on different stages of follicles in ovaries removed from adult female BALB/c mice (*n* = 6). (C) The ratio of primary follicles to primordial follicles in different groups (*n* = 6). This ratio was 1 for the control group; the data in the CRS group were standardized by the ratio of the control group, and then the odds ratios were calculated and analyzed statistically. (D) The serum AMH level of mice in both the CRS (*n* = 10) and control groups (*n* = 15). (E)The mRNA expression level of AMH in mouse ovaries of both the CRS and control groups (*n* = 6) (*P< 0.05, **P< 0.01, ***P< 0.001, ****P< 0.0001). CRS, Chronic restraint stress.

The H&E staining of ovarian sections was performed at 3 and 8 weeks after stress to evaluate the morphological structure of the ovaries (Fig 2A). Moreover, follicles in different stages including primordial follicles, primary follicles, secondary follicles, and corpus luteums were counted. Statistical analysis results showed that 8 weeks of CRS induced a significant decrease in the number of primordial follicles (Fig 2B, *P*< 0.01), while the number of primary follicles significantly increased in the CRS 8w group (Fig 2B, *P*< 0.0001). Moreover, the ratio of primary follicles to primordial follicles was significantly higher in both the CRS 3w and CRS 8w groups compared with that in the control group (Fig 2C, *P*< 0.01 and *P*< 0.0001, respectively). These results implied a wave of activation of dormant primordial follicles in the CRS group, and this situation was more notable after a longer period of CRS treatment. Also, the numbers of secondary follicles and corpus luteum were also reduced in the CRS group (Fig 2B).

Anti-Mullerian hormone (AMH) is a novel indicator of ovarian reserve. The level of AMH is in line with the number of small, growing follicles in the ovary, including primary follicles [8, 29]. The AMH levels were detected in both the serum and ovaries of mice by ELISA and PCR. A sharp increase to nearly twofold in the level of serum AMH was observed in the CRS 8w group compared with that in the control group (Fig 2D, P< 0.0001). Furthermore, the mRNA expression level of Amh increased significantly in the ovarian tissue of CRS 8w group (Fig 2E, P< 0.01). These results indicated that the number of small, growing follicles significantly increased in the CRS 8w group, which was consistent with the follicle counting results.

### CRS induced the overexpression of Kitl and activation of PI3K/PTEN/Akt pathway

**Fig 3:**
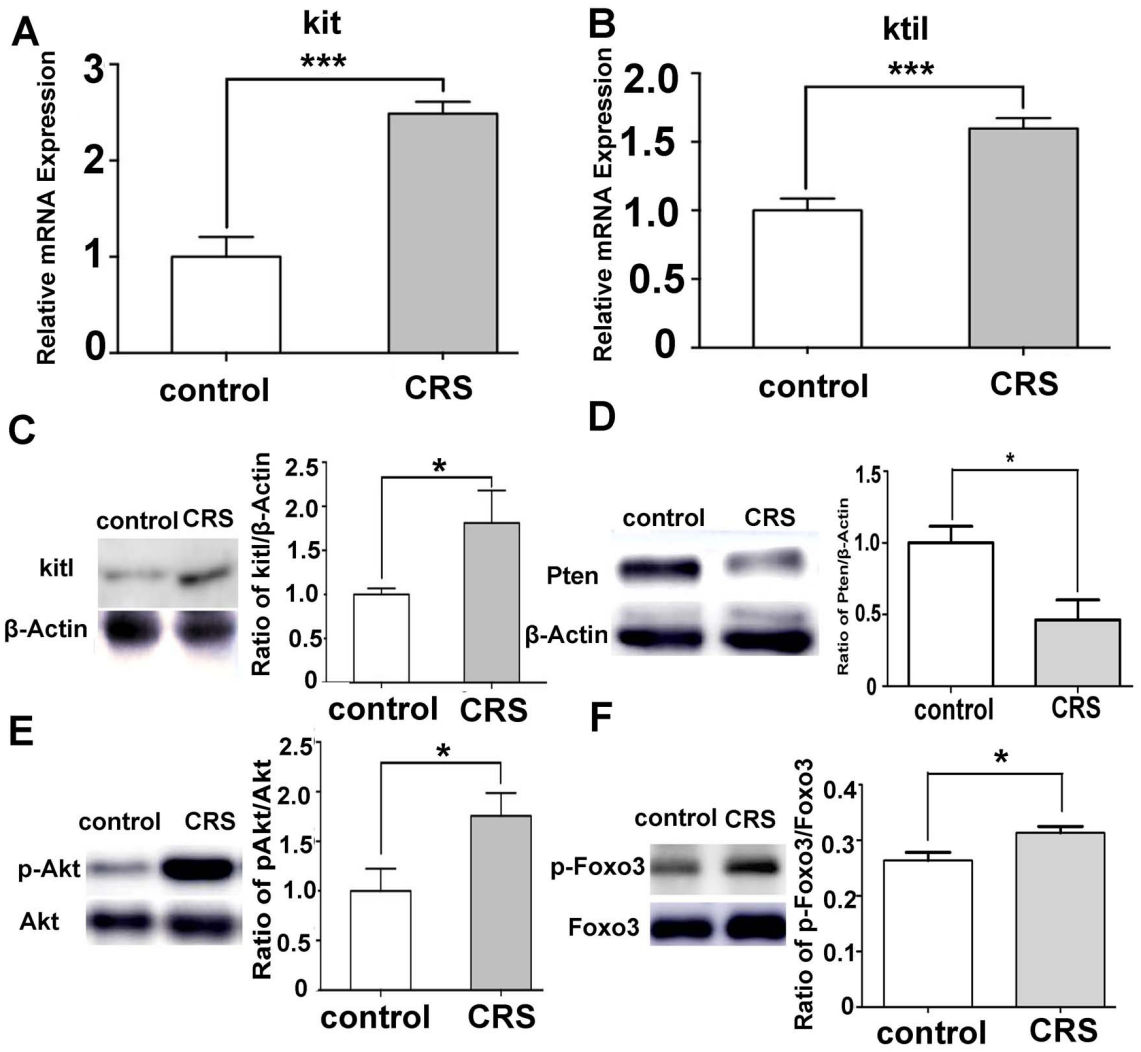
CRS increased the expression of Kitl/Kit and triggered the activation of PTEN/PI3K/Akt pathway. (A, B) The mRNA expression level of Kit and Kitl in mouse ovaries of both CRS and control groups (*n* = 6, *P*< 0.001). (C) The protein expression level of Kitl in the ovaries of the CRS group was significantly higher compared with that in the control group (*n* = 6, *P*< 0.05, normalized according to the b-Actin values). (D) The expression level of PTEN decreased significantly in the ovaries of the CRS 8w group (*n* = 6, *P*< 0.05). (E) The ratio of pAkt protein expression level to Akt values in the ovaries of the CRS group was significantly higher compared with that in the control group (*n* = 6, *P*< 0.05). (F) The ratio of pFoxo3 protein expression level to Foxo3 values in the ovaries of the CRS group was significantly higher compared with that in the control group (*n* = 6, *P*< 0.05). CRS, Chronic restraint stress.

The PI3K/PTEN/Akt pathway plays an essential role in the activation of primordial follicles, which can be activated by Kitl/Kit signaling [14,15,19]. Since the Kitl -PI3K signaling pathway has been indicated to play a central role in the activation of primordial follicles, it was hypothesized that this signaling pathway might participate in the activation of primordial follicles induced by stress in the CRS mouse model. Real-time PCR and Western blot showed that the expression levels of both Kitl and its oocyte receptor Kit were significantly elevated in the ovarian tissues of CRS 8w group compared with that of the control mice (Fig 3A, *P*< 0.001; Fig 3B, *P*< 0.001; Fig 3C, *P*< 0.05). Moreover, the expression level of *PTEN*, the major inhibitor of this pathway, decreased significantly in the ovaries of the CRS 8w group (Fig 3D, *P*<0.05). The Western blot analysis showed that Akt and Foxo3 were highly phosphorylated in the ovaries of the CRS 8w group (Fig 3E and 3F, *P*< 0.05).

**Fig 4:**
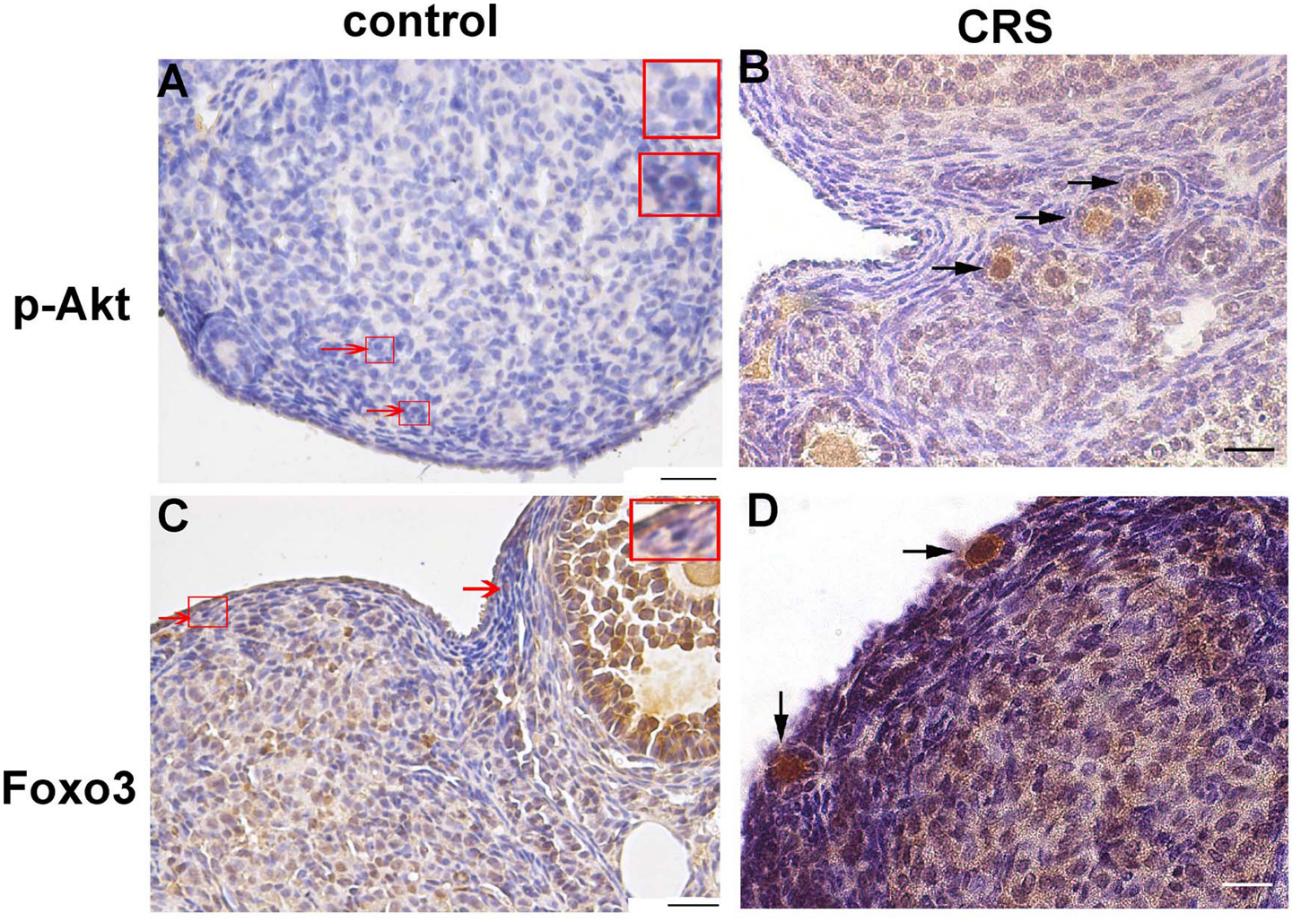
Immunohistochemical staining of pAkt and Foxo3 in the ovaries of the control and CRS 8w groups. The staining of pAkt and Foxo3 was obviously observed in the cytoplasm of oocytes in primary follicles of the CRS 8w group compared with that of the control group ((red arrows pointing to primordial follicles and black arrows to primary follicles; scale bar, 50 μm). CRS, Chronic restraint stress.

Further, the immunohistochemical analysis confirmed the highly activated states of the downstream PI3K/PTEN/Akt pathway. More primary follicles were observed, which were positively stained with pAkt in the ovaries of the CRS 8w group. However, more unstained primordial follicles were observed in the ovaries of the control group. Moreover, Foxo3, as an essential transcription factor, marked the activation of primordial follicles, which were obviously observed in the cytoplasm of oocytes in the primary follicles of the CRS 8w group (Fig 4).

### CRH promoted activation of primordial follicles in the cultured ovaries of newborn mice

**Fig 5:**
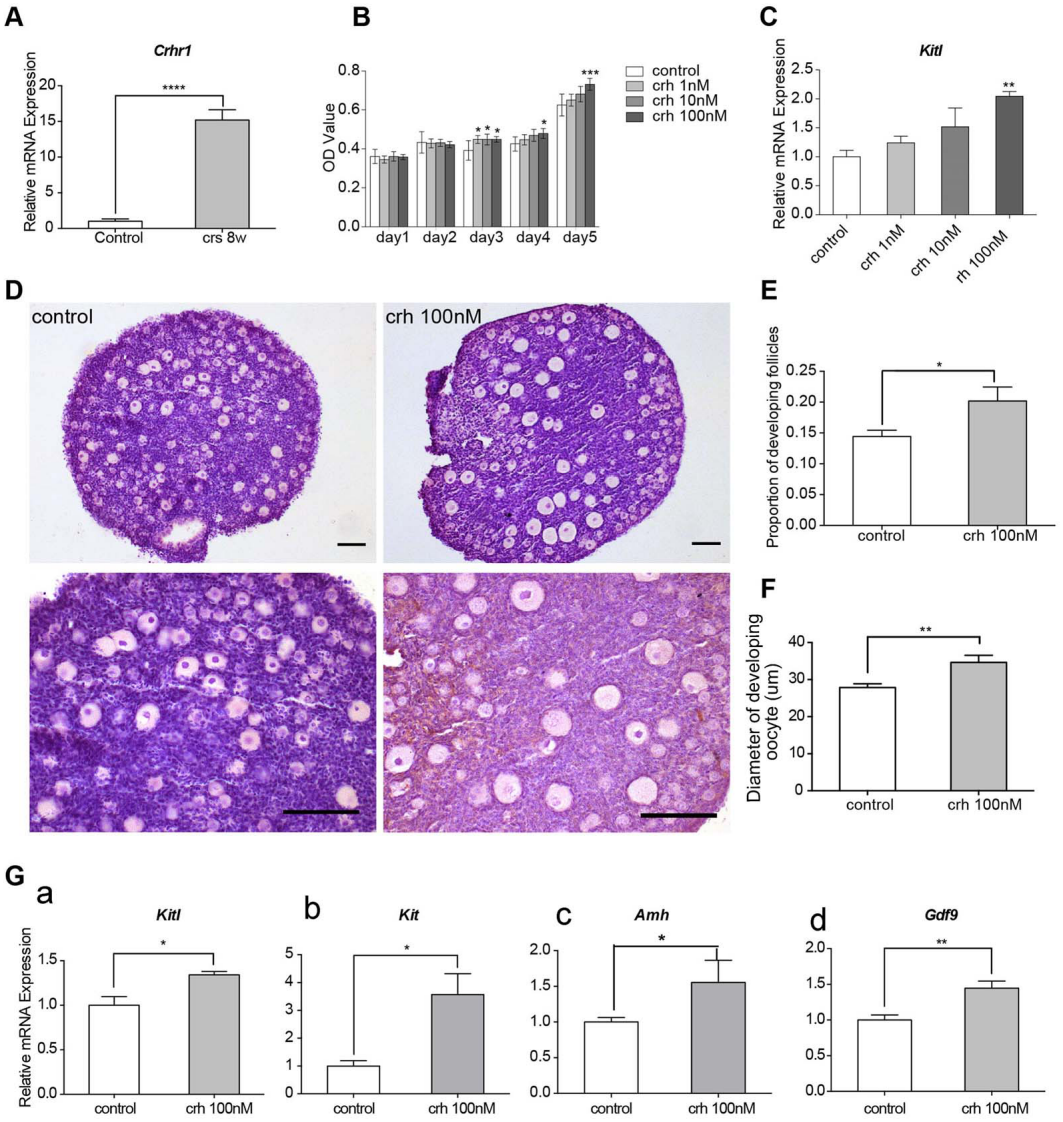
CRH triggers the activation of primordial follicles in the cultured ovaries of newborn mice *in vitro*. (A) The mRNA expression level of CRH receptor CRHR1 in the ovaries of the CRS group was significantly higher compared with that in the control group (*n* = 6). (B) The cell viability of mice granulosa cells (GCs) increased with the treatment of increasing concentration of CRH using theCCK-8 assay (*n* = 12). (C) The mRNA expression level of Kitl in mice GCs after culturing in serial concentration of CRH for 48 h (*n* = 6). (D) H&E staining of cultured mice ovaries from the control or CRH groups. Ovaries were isolated from the 3-day postnatal mice and cultured *in vitro* with or without CRH (100nM) for 7 days (scale bar, 100 μm). (E) CRH treatment increased the proportion of developing follicles in cultured mice ovaries (*n* = 6). (F) CRH treatment increased the diameter of oocytes in developing follicles in cultured mice ovaries (*n* = 6). (G) The mRNA levels of Kitl, Kit, AMH, and Gdf9 in cultured mice ovaries increased after CRH treatment (*n* = 6). (*P<0.05,**P< 0.01,***P< 0.001,****P< 0.0001).

This study focused on the functions of stress-related hormones to elucidate the link between stress responses and the effect on activation of primordial follicles. Among the hormones studied, CRH is a neuropeptide caused by stress, which also played an important role in regulating ovarian functions and follicle development[30–32]. It was found that the serum CRH level in the CRS 8w group was significantly higher compared with that in the control group (Fig 1F). Further, a real-time PCR assay showed that the mRNA level of its main receptor *Crhr1* dramatically increased in the ovaries of the CRS 8w group compared with that in the control group (almost 14-fold higher; Fig 5A, *P*< 0.05).

Serial concentrations of CRH were added to the *in vitro* culturingmice granulosa cells to further explore whether CRH was related to excessive activation of primordial follicles after CRS. The CCK-8 assay showed that a higher concentration of CRH significantly promoted cell viability and proliferation (Fig 5B, *P*< 0.05). After 48 h of culture, the mRNA level of *Kitl* significantly increased under treatment with 100nM CRH (Fig 5C, *P*< 0.05).

Then, an *in vitro* mouse ovary culture system was adopted to assess how CRH affected the activation of primordial follicles. In brief, ovaries were dissected from 3-day-old mice and placed in control or 100nM CRH conditions. After culturing for 7 days, the ovaries were collected for H&E staining and real-time PCR assay. As shown in Fig 5D–5F, the diameters of developing oocytes in 100nM-CRH-treated ovaries were significantly greater than those in the control group (*P*<0.01), and the proportion of developing follicles was also higher in the 100nM-CRH-treated group (*P*< 0.05).

Furthermore, the expression mRNA levels of genes highly associated with the follicular development were evaluated in the culturing ovaries with or without CRH. The results showed that the mRNA expression levels of *Kitl*, *Kit*, *Amh*, and *Gdf9*genes increased significantly under the stimulation of CRH (Fig 5Ga–5Gd, *P*< 0.05, *P*< 0.05, *P*< 0.05, and *P*< 0.01, respectively).

## Discussion

Stress is an inevitable part of modern life. Since stress can alter immunological, neurochemical, and endocrine functions of the bodies, it is known that stress can have detrimental effects on multiple diseases. It is believed that the HPA axis, when activated by stress, exerts an inhibitory effect on the female reproductive system [20]. The CRS model of mice was adopted in this study. After 8 weeks of chronic restraint treatment, it was found that the estrous cycles of mice were completely disordered. The results of follicle counting suggested that CRS affected regular ovulation, since the number of corpus luteum significantly decreased after stress. The number of secondary follicles also decreased, suggesting that stress delayed further development of primary follicles. Most importantly, a reduction in primordial follicles and an increment in primary follicles were observed in the ovaries of the CRS group. This trend started to occur after 3-week restraint and became significant in 8-week restraint group, indicating that stress induced a wave of primordial follicle loss in these CRS animal models.

The activation of primordial follicles is a highly regulated process, whose underlying mechanisms are still not fully interpreted [12, 33]. A previous study showed that the GCs of primordial follicles play a fundamental role in initiating the activation of primordial follicles. An elevated expression of Kitl can activate the PI3K pathway, turning oocytes into an awaking status [19]. This study found that the levels of Kitl and its oocyte receptor Kit were both elevated in the CRS-treated mouse ovaries. The Akt protein was highly phosphorylated in the ovaries of the CRS 8w group, and the level of inhibition protein PTEN decreased. Therefore, it was suggested that the activation of Kitl/Kit signaling and PI3K/PTEN/Akt pathway could account for the activation of primordial follicles occurring in the CRS-treated mice. However, the alteration of Kitl/Kit–PI3K/PTEN/Akt signaling pathway might also be the consequence of activation of primordial follicles. Also, the PI3K signaling pathway highly and broadly regulated cell survival and proliferation [34,35]. A high level of Akt phosphorylation was observed inside the oocyte nucleus of primary follicle in the ovaries of the CRS group mice using immunohistochemistry staining, and the results of Western blot further confirmed the Akt phosphorylation level of the whole ovary of CRS mice.

Studies have shown that the Kitl/Kit system in the ovary does not solely influence the follicle growth and maturation [36, 37]. In the ovary, theca cells arise when a follicle transitions from its growth phase and begins to produce steroid hormone. Kitl/Kit interactions have been found to be essential in promoting follicular development to the primary follicle stage in mice. Kitl plays an organizer role in inducing stromal cells to differentiate into theca cells expressing steroidogenic enzymes and LH receptors [38, 39]. Moreover, the theca cells contribute critically to the transformation of the follicular wall into the compact and highly vascular corpus luteum following ovulation. The Kit-expressing cells contribute to the complicated network of capillaries and small luteal cells that surround the large luteal cells, suggesting that Kitl/Kit interactions may be important in regulating the lifespan and function of luteal cells[40, 41]. However, follicle counting in this study showed a decline in the number of both secondary follicles and corpus luteum in the CRS group. Whether the decline in the number of corpus luteum is related to the upregulation of Kitl/Kit induced by excessive activation of primordial follicles needs to be further elucidated.

PTEN, known as a tumor suppressor and negative regulator of the PI3K/Akt pathway, was involved in follicular development. The PTEN loss mouse model showed that a selective homozygous loss of PTEN in early follicle oocytes, but not in oocytes of later stage (primary and beyond) follicles, caused premature activation of the primordial follicle pool and premature ovarian failure [42, 43]. Consistent with these, 8 weeks of CRS induced excessive activation of primordial follicles in female mice, accompanied by the upregulated expression of p-Akt and downregulated expression of PTEN. However, studies have found that the global loss of PTEN increased the number of corpus luteum, enlarged the ovarian size, and prolonged the lifespan of corpus luteum with disrupted luteolysis[44–46]. The weight of ovaries and number of corpus luteum decreased in the CRS group, however, whether it is related to the down-regulation of PTEN needs to be further elucidated.

CRH was originally recognized as a neuropeptide secreted from the hypothalamus, which plays an important role in response to stress. However, available evidence has shown that CRH tends to regulate multiple reproductive functions, including ovulation, luteolysis, decidualization, implantation, and early maternal tolerance [20]. In this study, CRS induced the increasing levels of serum corticosterone and CRH in the CRS mouse model. CRHR1 is a receptor of CRH, which exists abundantly in ovaries, including GCs, theca cells, and corpus luteum [47, 48]. An *in vitro* ovary culture system was used to further investigate the relationship between stress and activation of primordial follicles. Ovaries from newborn mice mostly contain newly formed primordial follicles; therefore, they are appropriate models for studying the progression in the activation of primordial follicles. In this study, 100nMCRH was added into the culture system, which was a level above the physiological concentration in mice. It was found that this higher level of CRH could up-regulate the expression of Kitl in both human GCs and cultured newborn ovaries. Moreover, CRH induced a wave of activation of primordial follicles in the newborn mouse ovaries compared with the control group. Thus, it was concluded that CRH, which was abundantly secreted in stress conditions, might be responsible for the excessive activation of primordial follicles caused by chronic stress. However, this study had certain limitations. First, the concentration of CRH to be used in the *in vitro* experiment had to be decided. One study showed that the CRH concentration in a mouse ovary could be more than 30 times higher than that in the serum [48]. Other studies used different concentrations of CRH ranging from 1nM to 100nM to evaluate the effect of CRH on the *in vitro* culture of GCs or on the *in vitro* growth of preantral follicles and early embryo development [32, 49]. Therefore, the serial concentration of CRH was used to observe the effect of CRH on the cellular proliferation of human GCs and the activation of follicles of newborn ovaries.

Moreover, stress can also raise the levels of other hormones *in vivo*, such as adrenocorticotropic hormone or glucocorticoids [50–52]. Therefore, to further verify whether CRH is the true endocrine factor for the activation of primordial follicles, more experiments, such as using antalarmin, a nonpeptide drugthat blocks the CRHR1, are needed.

AMH is a member of the transforming growth factor-β family. The measurement of peripheral AMH was applied to a wide range of clinical applications, mainly based on its ability to reflect the number of small, growing follicles present in the ovaries [29, 53, 54]. In the present study, a sharp increase was found in the number of primary follicles after 8 weeks of CRS treatment, which correlated with the significantly elevated AMH levels in both mouse serum and ovaries. In physiological situations, the activation and resting of primordial follicles were regulated by multiple signaling in local microenvironment and maintained a certain balance [53]. Herein we think CRS might interrupt the balance of activation and resting of primordial follicles, causing excessive primordial follicle development, and resulting in the elevation of AMH levels in both the ovary and blood serum.

It is essential that a certain number of the dormant primordial follicles stay in a quiescent state to provide a continuous supply of developing follicles to maintain the length of the reproductive span in women [55]. As the primordial follicles are recruited shortly after birth, only relatively fixed reservation is preserved in the ovaries. In theory, menopause occurs when this fixed follicle pool finally exhausts. Such situation in women under age 40 years is determined as premature ovarian insufficiency(POI) clinically [56]. However, its etiology remains unknown in approximately 90% cases [57]. Bleilet al investigated a study of 979 participants of ages 25–45 women and found that psychological stress was related to higher antral follicle count(AFC) among younger women and to a higher rate of AFC decline across women, suggesting that greater stress may enhance reproductive readiness in the short term at the cost of accelerating reproductive aging in the long term (58). Consistent with it, our study provides an experiment evidence that a long period of psychological stress could cause excessive activation of primordial follicles, which might in turn contribute to the depletion of primordial follicles and early exhaustion of ovarian reservation.

## Conclusions

**Fig 6:**
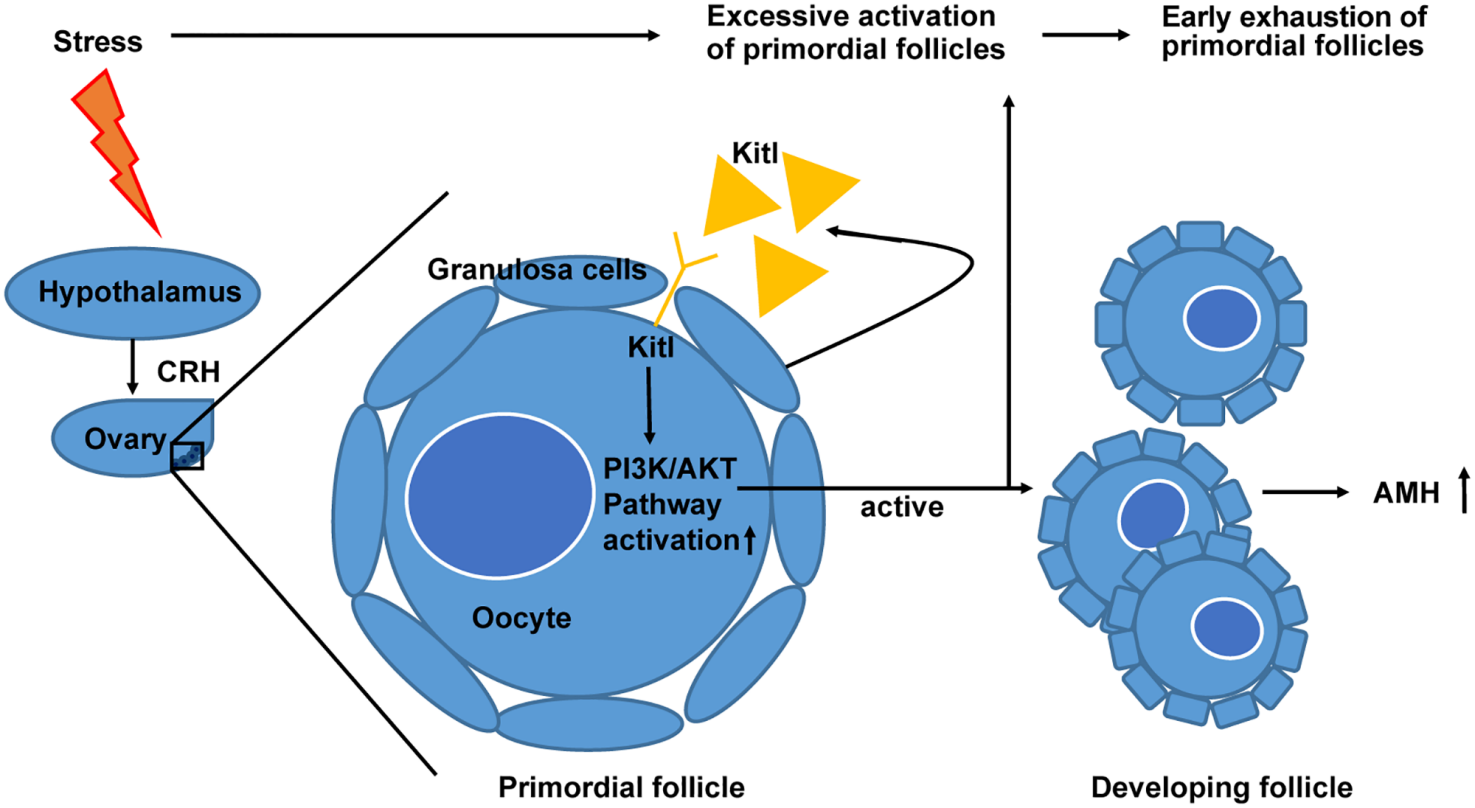
Schematic diagram showing the course of CRS inducing abnormal activation of primordial follicles. CRS leads to the excessive activation of primordial follicles, accompanied by up-regulated expression of Kitl and its receptor Kit, and the activation of PI3K/PTEN/Akt pathway. What is more, the level of serum AMH was increased significantly after CRS. CRH induced by chronic stress might play an important role in the activation and exhaustion of primordial follicles. CRS, Chronic restraint stress.

In conclusion, as illustrated in Fig 6, this study demonstrated that 8 weeks of CRS in female BALB/c mice led to the excessive activation of primordial follicles, accompanied by up-regulated expression of Kitl and its receptor Kit, and the activation of PI3K/PTEN/Akt pathway. CRH induced by chronic stress might play an important role in the activation of primordial follicles.

## Supporting information

S1 Fig (A) CRS did not affect food and water intake of mice (*n* = 6, P> 0.05). (B, C) Serum concentration of estradiol and testosterone in mice of both control and CRS 8w groups. CRS, Chronic restraint stress.
(TIF)

S1 Table PCR primer sequences. (DOC)
