# Supplementary figures and images for: Chronic restraint stress induces excessive activation of primordial follicles in mice ovaries
Source: PLoS One. 2018 Mar 30;13(3):e0194894. doi: 10.1371/journal.pone.0194894 (PMC5877864; doi:10.1371/journal.pone.0194894)

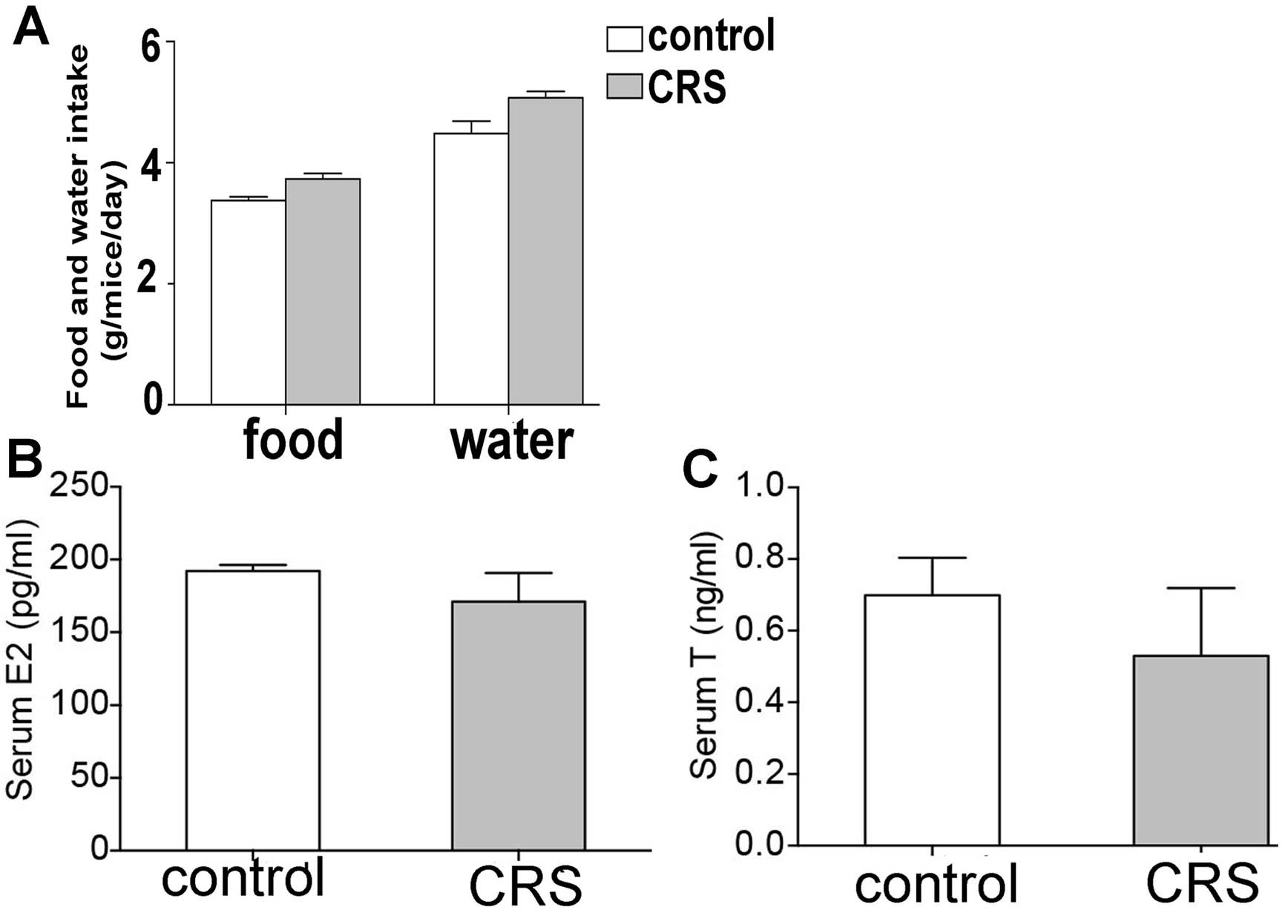

Supplement: S1 Fig — (A) CRS did not affect food and water intake of mice (n = 6, P> 0.05). (B, C) Serum concentration of estradiol and testosterone in mice of both control and CRS 8w groups. CRS, Chronic restraint stress. (TIF) [file pone.0194894.s001.tif]
