# Supplementary material for: Chronic restraint stress induces excessive activation of primordial follicles in mice ovaries
Source: PLoS One. 2018 Mar 30;13(3):e0194894. doi: 10.1371/journal.pone.0194894 (PMC5877864; doi:10.1371/journal.pone.0194894)
Supplement: S1 Table — (DOC) [file pone.0194894.s002.doc]

**STable1.** PCR primer sequences.

| Gene | Primer sequence 5’→3’ | | Amplicon size (bp) |
| --- | --- | --- | --- |
| mouse Kit | Forward | CTGGGATCTGCTCTGCGTC | 167 |
|  | Reverse | AGTCGGGATCAATGCACGTC |  |
| mouse kitl | Forward | AGAAGACACAAACTTGGATTATCAC | 183 |
|  | Reverse | CATCCATCCCGGCGACATAG |  |
| mouse Αmh | Forward | GCAGGCCCTGTTAGTGCTAT | 197 |
|  | Reverse | TCAGGGTGGCACCTTCTCTG |  |
| Mouse  GDF9 | Forward | CCTCTACAATACCGTCCGGC | 115 |
|  | Reverse | CACCCGGTCCAGGTTAAACA |  |
| Mouse  CRHR1 | Forward | GGACAAGCACTCCATCAGAG | 230 |
|  | Reverse | CAGGCTTACAGGTCTGCATC |  |
| Mouse  β-actin | Forward | CGCAGCCACTGTCGAGT | 194 |
|  | Reverse | CCCACGATGGAGGGGAATAC |  |
